# Advancing tuberculosis diagnosis and management in cynomolgus macaques using Xpert MTB/RIF ultra assay

**DOI:** 10.1038/s41598-024-51824-y

**Published:** 2024-01-17

**Authors:** Prapaporn Srilohasin, Saradee Warit, Therdsak Prammananan, Saijai Smithtikarn, Reka Kanitpun, Mutchamon Kaewparuehaschai, Kirana Noradechanon, Suthirote Meesawat, Nattakan Thakaew, Nonthakorn Sakulwittayasuk, Taratorn Kemthong, Tanapat Palaga, Suchinda Malaivijitnond, Angkana Chaiprasert

**Affiliations:** 1 Department of Microbiology, Faculty of Medicine Siriraj Hospital, Mahidol University, Bangkok, Thailand; 2 Drug Resistant Tuberculosis Research Fund, Siriraj Foundation, Bangkok, Thailand; 3 National Center for Genetic Engineering and Biotechnology, National Science and Technology Development Agency, Khlong Luang, Thailand; 4 Division of Tuberculosis, Department of Disease Control, Ministry of Public Health, Bangkok, Thailand; 5 National Institute of Animal Health, Bangkok, Thailand; 6 Department of National Parks, Wildlife and Plant Conservation, Chachoengsao, Thailand; 7 National Primate Research Center of Thailand-Chulalongkorn University, Saraburi, Thailand; 8 Department of Microbiology, Faculty of Science, Chulalongkorn University, Bangkok, Thailand; 9 Office for Research, Faculty of Medicine Siriraj Hospital, Mahidol University, Bangkok, Thailand

**Keywords:** Infectious-disease diagnostics, Microbiology techniques

## Abstract

The detection and management of *Mycobacterium tuberculosis* complex (MTBC) infection, the causative agent of tuberculosis (TB), in macaques, including cynomolgus macaques (*Macaca fascicularis*), are of significant concern in research and regions where macaques coexist with humans or other animals. This study explored the utility of the Xpert MTB/RIF Ultra assay, a widely adopted molecular diagnostic tool to diagnose tuberculosis (TB) in humans, to detect DNA from the *Mycobacterium tuberculosis* complex in clinical samples obtained from cynomolgus macaques. This investigation involved a comprehensive comparative analysis, integrating established conventional diagnostic methodologies, assessing oropharyngeal-tracheal wash (PW) and buccal swab (BS) specimen types, and follow-up assessments at 3-month, 6-month, and 12-month intervals. Our results demonstrated that the Xpert MTB/RIF Ultra assay was able to detect MTBC in 12 of 316 clinical samples obtained from cynomolgus macaques, presenting a potential advantage over bacterial culture and chest radiographs. The Xpert MTB/RIF Ultra assay exhibited exceptional sensitivity (100%) at the animal level, successfully detecting all macaques positive for *M. tuberculosis* as confirmed by traditional culture methods. The use of PW samples revealed that 5 positive samples from 99 (5.1%) were recommended for testing, compared to 0 samples from 99 buccal swab (BS) samples (0.0%). In particular, the definitive diagnosis of TB was confirmed in three deceased macaques by MTB culture, which detected the presence of the bacterium in tissue autopsy. Our findings demonstrate that the implementation of the Xpert MTB/RIF Ultra assay, along with prompt isolation measures, effectively reduced active TB cases among cynomolgus macaques over a 12-month period. These findings highlight the advance of the Xpert MTB/RIF Ultra assay in TB diagnosis and its crucial role in preventing potential outbreaks in cynomolgus macaques. With its rapidity, high sensitivity, and specificity, the Xpert MTB/RIF Ultra assay can be highly suitable for use in reference laboratories to confirm TB disease and effectively interrupt TB transmission.

## Introduction

*Mycobacterium tuberculosis* is the primary causative agent responsible for the global occurrence of tuberculosis (TB) in humans, contributing to an annual death toll of 1.6 million^1^. However, TB can also inadvertently infect animals. Our understanding of tuberculosis in animals, particularly cynomolgus macaques (*Macaca fascicularis*; Mf), is restricted, despite their frequent cohabitation with humans in various regions and their application as models of nonhuman primate (NHP) to investigate infectious diseases of humans^2^. The demand for improved diagnostic, preventive, and therapeutic strategies to combat tuberculosis (TB) in cynomolgus macaques is increasing steadily^3^. Although tuberculin skin testing (TST) and chest radiographs are common diagnostic methods valuable in TB diagnosis among cynomolgus macaques, they possess limitations that can lead to nonspecific results, primarily due to exposure to non-TB bacteria or other factors. Cross-reactivity with non-TB mycobacteria can result in false positives. Interferon-Gamma Release Assays (IGRA), used to diagnose tuberculosis (TB) infection, are not without controversy and limitations when used in macaques^4,5^. These limitations include the complexity of immune responses in macaques, variations in the interpretation of results, and the limited sensitivity of IGRAs in the detection of latent infections. Microbiological culture and molecular assays are widely recognized as definitively confirming TB disease. These approaches offer a high level of reliability by directly detecting the presence of TB-causing bacteria or their genetic material. The use of microbiological culture in cynomolgus macaque samples comes with a series of pitfalls and challenges, particularly in the context of tuberculosis. It is possible that TB bacteria do not exhibit growth within a laboratory environment or are overshadowed by the proliferation of other microorganisms. The sensitivity, specificity, and cost-effective rate of molecular assays that can identify bacteria even in small amounts are promising tools to diagnose TB in cynomolgus macaques. Molecular assays demonstrate an impressive capacity to detect bacteria, even in trace amounts, and offer attractive features, including high sensitivity, specificity, and cost-effectiveness. These attributes establish molecular assays as invaluable tools for diagnosing TB in cynomolgus macaques^2,6^. However, the application of molecular assays to diagnose tuberculosis in macaques does come with certain challenges. Factors such as the potential interference of inhibitors in assay accuracy, the risk that the amount of TB DNA in the sample falls below the detection threshold, the need for specialized laboratory expertise, and the possibility of cross-reactivity are all elements that can impact the reliability of these assays in this specific context.

The Xpert MTB/RIF Ultra assay, endorsed by the World Health Organization (WHO) as a primary diagnostic tool for human patients^7,8^, is a molecular diagnostic test that uses the real-time polymerase chain reaction (PCR) technique to detect the DNA of the *Mycobacterium tuberculosis* complex (MTBC). It also identifies mutations associated with resistance to rifampicin (RIF), directly from patient sputum and other clinical samples. A prospective multicenter study conducted on adults with pulmonary tuberculosis revealed that the Xpert MTB/RIF Ultra test addresses several limitations of traditional TB diagnostic methods and offers faster, more accurate, performance in detecting MTBC DNA in extrapulmonary samples, and a user-friendly approach to detect *M. tuberculosis* complex DNA and resistance mutations of RIF^9,10^. The performance of the Xpert MTB/RIF Ultra assay has the potential to significantly enhance the speed and accuracy of the diagnosis of tuberculosis in macaques. This advance could enable timely decisions to interrupt transmission and contribute to more effective management of tuberculosis in these animals.

Long-tailed macaques (Mf) are prevalent in various parts of Thailand^11^, often found in areas where human communities overlap with their natural habitats. These macaques, protected by Thai law, have been captured, conserved, and cared for at Wildlife Rescue Centers and, in certain cases, rescued from illegal captivity. The Krabok-Koo Wildlife Breeding Center is one of the proactive facilities with dedicated staff who prioritize the well-being of these animals without neglect. A previous study reported tuberculosis (TB) cases in this center^4^, underlining the urgency of effective tools for the detection of TB and concerns for the welfare of these animals, given their potential as a model.

## Methods

### Animals and specimen collection

**Figure 1:**
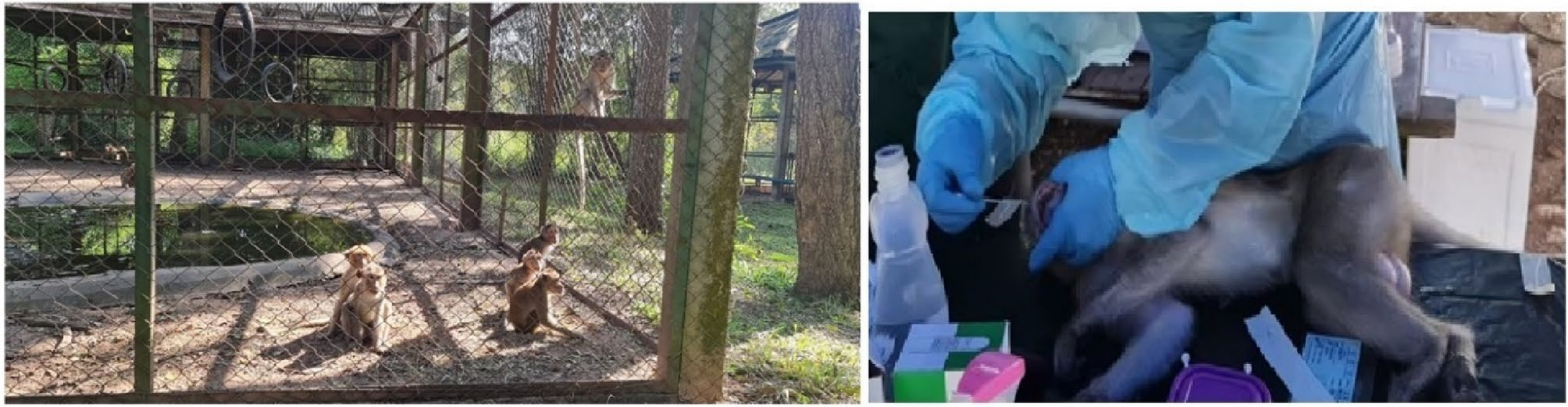
Cynomolgus monkeys in a realistic social context and sample collection.

This study constitutes a prospective cohort investigation that was conducted at the Krabok-Koo Wildlife Breeding Center, Tha Takiap District, Chachoengsao Province, Thailand. Research was carried out in a cohort of captive cynomolgus macaques, in total 316 individuals distributed in 42 group cages, each accommodating 5–13 animals. The cages were exposed to the prevailing natural environmental onditions (Fig. 1). The adjacent cages are placed at a minimum separation of 10 m from each other. Among cynomolgus macaques, 250 (79.1%) were identified as males, while 276 (84.7%) exhibited an initial body weight greater than 4 kg, with a weight range ranging from 2 to 10 kg. The estimated age based on dental eruption patterns^12^ of the macaques ranged from 2 to 15 years old. Before undergoing various research procedures, the cynomolgus macaques were subjected to anesthesia using a mixture of Zoletil® (3–5 mg per kg) and dexmedetomidine hydrochloride (0.03–0.05 mg per kg). This anesthesia protocol ensured their safety and comfort during essential procedures, including collection of PW samples (Fig. 1), BS, body weight recording, tuberculin skin test administration (TST), acquisition of chest radiographs and other necessary physical examinations. Throughout the course of the study, these animals will be meticulously monitored at regular three-month intervals, during which body weight recording, chest radiographs, specimen collection for culture, antibody detection and IGRA and other necessary physical examinations will be examined, allowing for comprehensive observation and data collection. For suspected or confirmed cases of infectious TB, the macaques were immediately relocated to isolation cages located a significant distance from their original housing. The experiments and methods were approved by the Department of National Parks, Wildlife and Plant Conservation, Thailand (Animal Ethics No. 012/2564, approved by Mahidol Committee, Mahidol University Protocol review No. 009/2564). All methods were also performed in accordance with the relevant guidelines and regulations. All monkeys were handled by the Wildlife Rescue Center No.2 (Krabokkoo) veterinarians and capture teams according to their guidelines. Additionally, this study was conducted in compliance with the Animal Research: Reporting of In Vivo Experiments (ARRIVE) guidelines.

### Specimen preparation and testing with Xpert MTB/RIF ultra-assay

To achieve our research objectives, the 316 PW samples from cynomolgus macaques underwent thorough analysis using the Xpert MTB/RIF Ultra assay, a method designed for the precise identification of the DNA of the *M. tuberculosis* complex. The assay employs real-time polymerase chain reaction (PCR) technology to amplify and detect specific regions of the MTBC genome, with a focus on the insertion sequence (IS) *6110*, IS*1081* element and RNA polymerase β subunit (*rpoB*) gene. Real-time monitoring of fluorescence melting curve patterns allows for continuous detection of present MTBC and RIF resistance. Furthermore, we conducted follow-up evaluations at 3-month, 6-month, and 12-month intervals for a total of 99, 95, and 87 cases, respectively (Annex 1). To investigate the potential of Xpert MTB/RIF Ultra to detect the presence of MTBC DNA in PW and BS samples of cynomolgus macaques, 99 paired samples were tested.

PW involved collecting samples from the upper respiratory tract, specifically the oropharyngeal (mouth and throat) and tracheal (windpipe) areas. And BS involves the use of a sterile cotton swab to collect cells from the inside of the cheek (buccal mucosa). Regarding the Xpert MTB/RIF Ultra assay, 1 ml of PW or resuspended BS was transferred to a sealed sterile tube containing 2 ml of sample reagent. The mixture was vigorously shaken for a minimum of 10 s and subsequently incubated at room temperature for 10 min. After this initial incubation, the solution was subjected to further rigorous agitation and then rested for 5 min. The supernatant was gently transferred to the reaction cartridge using the provided transfer pipette. The entire testing procedure was completed in 90 min.

### Mycobacterial culture and species identification

The processing of PW samples was carried out for semiquantitative assessment (acid-fast bacilli (AFB) smear) and isolation purposes for all cases using the NaOH-NALC method^13^. Briefly, 1 ml of PW was mixed with an equal volume of 2% NaOH-NALC solution (consisting of 4% sodium hydroxide, 2.9% sodium citrate and 0.5% *N*-acetyl L cysteine) using a vortex mixer and incubated at room temperature for 15 min. Subsequently, 40 ml of sterile 0.067 M phosphate buffer (pH 7.0) were added, mixed by inverting 3–4 times, and centrifuged at 3000 × g for 20 min at 4 °C. The supernatant was then discarded and the pellets were resuspended with 1 ml of sterile 0.067 M phosphate buffer. Samples of 0.1 ml, 0.5 ml and 1 drop of the resuspended samples were added to Löwenstein Jensen Medium (LJ medium), MGIT media, and slides for AFB staining, respectively. Culture medium was incubated at 37 °C for four to eight weeks. AFB staining was performed using the Kinyoun technique in both sediment-positive and culture-positive samples. In-house multiplex PCR method^14^ and/or the GenoType Mycobacterium CM kit (Hain Life Science, Germany) were used to test AFB-positive colonies for identification of the presence of Mtb.

### Statistical analyzes

The overall positive rates between PW and BS were compared using the McNemar test. The analyzes were performed using SPSS software version 18.0 (SPSS Inc., Chicago, USA).

## Results

### Evaluation of the Xpert MTB/RIF ultra assay for the diagnosis of tuberculosis in cynomolgus macaques: a comparative analysis with conventional methods

In this study, we applied one of the most esteemed diagnostic tools available for tuberculosis diagnosis in human patients, and transposed it onto cynomolgus macaques. A bacterial culture assay was employed as the assay control. A comprehensive analysis of 316 different PW samples was conducted. The Xpert MTB/RIF Ultra assay successfully identified tuberculosis in 12 of these samples, accounting for 3.8% of the total samples. Among these cases, when we used the culture method combined with the GenoType Mycobacterium assay (Hain Lifescience GmbH, Nehren, Germany) for testing, 1 sample (8.3%) showed positive results for *M. tuberculosis*, despite that 8 other samples (66.7%) had positive AFB staining mixed with other bacteria. These samples comprised *M. intracellulare* (n = 1, 8.3%), *Mycobacterium* sp. (n = 2, 16.7%), *M. fortuitum* group (n = 1, 8.3%), and substantial overgrowth by other bacteria (n = 4, 33.3%). The remaining 2 samples (16.7%) were contaminated with other bacteria, while 1 sample (0.3%) did not show bacterial growth.

Subsequent to initial diagnostic assessments, a follow-up Xpert MTB/RIF Ultra testing regimen was conducted in 99 cynomolgus macaques that resided in 11 separate housing enclosures. Cumulatively, 16, 17 and 18 cases were diagnosed at 3, 6, and 12 months, respectively. Among the final 18 positive cases, 5 macaques died and tuberculosis was determined as the cause of death in 3 of these cases (Table 1). Overall, all macaques that were confirmed to be positive for *M. tuberculosis* by the culture method also tested positive with the Xpert MTB/RIF Ultra assay, demonstrating a 100% sensitivity of the method at the animal level (Table 1). Significantly, within the group of 41 TST positive cynomolgus macaques (13.0%), one individual tested positive for Xpert MTB/RIF Ultra three months later and unfortunately succumbed to tuberculosis 1 year later (Annex 1). An analogous observation was made within the subset of 42 cases (13.30%) exhibiting chest radiograph positivity. One case exhibited a positive outcome for the Xpert MTB/RIF Ultra assay 3 months after assessment, followed by the unfortunate outcome of tuberculosis-related mortality 1 year later. However, it should be noted that three of the twelve cases of Xpert MTB/RIF Ultra positive (25%) succumbed to tuberculosis 9 months after diagnosis. This outcome underscores the enhanced sensitivity and specificity of the Xpert MTB/RIF Ultra assay compared to TST and chest radiograph. And the test accurately detected the presence of MTBC DNA in PW samples, confirming the presence of the disease.

**Table 1:** Results of GeneXpert MTB/RIF Ultra, AFB, culture, and 12 month follow-up.

Mf no	Cage no	PW samples									Tissue autopsy
		Month 0		Month 3		Month 6		Month 9	Month 12		Alive/death (month)
		Xpert (Ct)	AFB, Culture	Xpert (Ct)	AFB, Culture	Xpert (Ct)	AFB, Culture	AFB, Culture	Xpert (Ct)	AFB, Culture	AFB, Culture
46	C1/1	+ (27.6)^T^	AFB + , Min	–	AFB + , cont	ND	ND	ND	ND	ND	Dead (7 MO), ND
49	C1/1	–	AFB + , Mfo	+ (23.7)^L^	NG	–	NG	NG	–	NG	Alive (9 MO)
55	C2/1	–	NG	+ (26.1)^L^	NG	–	NG	NG	–	NG	Alive (9 MO)
91	cage 5/1	+ (16.5)^L^	NG	ND	ND	ND	ND	ND	ND	ND	Dead (1 MO), ND
96	cage 5/1	+ (24.2)^T^	NG	ND	NG	–	NG	NG	–	NG	Alive (15 MO)
99	cage 5/1	–	NG	+ (19.9)^L^	NG	+ (17.2)^L^	AFB + , Mtb	ND	ND	ND	Dead (4 MO), Lung&Liver found Mtb
100	cage 5/1	–	NG	–	AFB−, cont	–	NG	NG	+ (17.0)^L^	NG	Alive (0 MO)
102	cage 5/1	+ (24.6)^T^	AFB + , Mtb	–	NG	–	NG	NG	–	NG	Alive (15 MO)
103	cage 5/1	+ (21.5)^T^	AFB + , cont	+ (17.9)^L^	NG	ND	AFB + , Mtb	ND	ND	ND	Dead (11 MO), Lung&Liver found Mtb
105	cage 5/1	+ (23.6)^T^	AFB + , Msp	–	AFB + , Msc	–	NG	NG	–	NG	Alive (15 MO)
120	cage 2/2	+ (25.2)^T^	AFB + , Mfo	–	AFB−, cont	–	NG	NG	–	NG	Alive (15 MO)
195	cage 4/5	+ (27.4)^T^	AFB + , cont	–	NG	–	NG	NG	–	NG	Alive (15 MO)
199	cage 5/2	+ (35.5)^T^	AFB–, cont	–	NG	–	NG	NG	–	NG	Alive (15 MO)
264	N9	+ (30.1)^T^	AFB + , cont	–	NG	–	NG	NG	–	NG	Alive (14 MO)
266	N9	+ (24.5)^T^	AFB–, cont	–	NG	–	NG	NG	–	NG	Alive (13 MO)
292	Q1	–	NG	–	NG	+ (26.1)^T^	NG	NG	–	NG	Alive (7 MO)
293	Q1	–	NG	+ (23.0)^T^	NG	+ (16.4)^M^	NG	ND	ND	ND	Dead (4 MO), Lung found Mtb
298	Q3	+ (29.3)^T^	AFB + , cont	–	NG	–	NG	NG	–	NG	Alive (13 MO)

+ , positive; -, negative; ND, not done; NG, No growth; cont, contaminate; Ct, cycle threshold; Min, *M. intracellulare*; Msc, *M. scrofulaceum*; Msp, *Mycobacterium s*p.; Mfo, *M. fortuitum*; Mtb, *M. tuberculosis*; MO, months; T, trace; L, Low; M, medium.

### Comparative analysis of oral-pharyngeal-tracheal wash and buccal swab specimens to detect MTBC DNA in cynomolgus macaques using the Xpert MTB/RIF ultra assay

To evaluate the types of specimens, PW and buccal swab (BS) were collected from the cynomolgus macaques follow-up group. The Xpert MTB/RIF Ultra assay was applied to a total of 99 paired specimens. Our comprehensive analysis revealed that the frequency of MTBC DNA detection was markedly elevated when using the Xpert MTB/RIF Ultra assay in PW samples, accounting for 5 out of 99 samples (5.1%), compared to BS samples, which did not produce positive detections out of 99 samples tested (0.0%). However, this divergence did not achieve a level of statistical significance as indicated by the data presented in Table 2 (*P* = 0.063). Of equal significance was the insight obtained from the subset of five positive macaques. Remarkably, one among them, animal numbered 103, demonstrated consistent positivity during subsequent rounds of tests (Table 1), implying the sustained presence of MTBC DNA. Intriguingly, three (animals numbered 99, 103, and 293) of the five positive macaques died in several months later. Bacteriological confirmation further validated these cases as definitive infections of tuberculosis (Table 1).

**Table 2:** Comparative Analysis of Xpert MTB/RIF Ultra Results between PW and BS Samples.

		No. (%) of BS result	
		Negative	Positive
No. (%) of PW result	Negative	94 (94.9)	0 (0.0)
	Positive	5 (5.1)	0 (0.0)

*P* = 0.063 by McNemar's test.

### Effective tuberculosis management and prevention strategies in a cynomolgus macaque population using the Xpert MTB/RIF ultra assay

**Figure 2:**
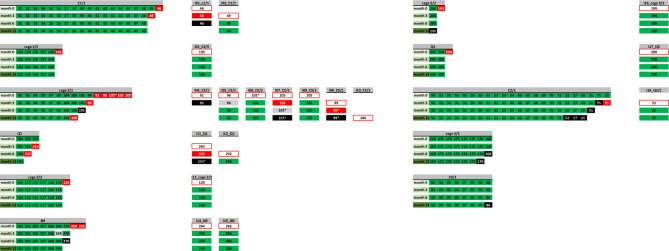
Mapping and summarizing the results obtained from the Xpert MTB/RIF Ultra assay among 100 cynomolgus macaques housed in 11 group cages: C1/1, cage 4/5, cage 5/1, Q1, cage 2/2, N9, cage 5/2, Q3, C2/1, cage 3/5 and C4/1, clustered at 3-month, 6-month and 12-month intervals. Each number corresponds to a cynomolgus ID. Negative results are represented in green, positive results are represented in red, and instances of death are indicated in black. An asterisk denotes cases that tested positive both in the Xpert MTB/RIF Ultra assay and in bacterial culture. Gray represents cases that were lost to follow-up. Cynomolgus macaques that were positive in the Xpert MTB/RIF Ultra assay were immediately relocated to new individual cages (i01–i08), as indicated by the open red rectangles.

Whenever an animal tested positive, it was immediately isolated to disrupt transmission and prevent further spread of the infection. Our results showed a decline in active TB cases over the 12 month period using the Xpert MTB/RIF Ultra assay (Fig. 2). Initially, 12 of the 316 cynomolgus macaques housed in 7 cages (C1/1, cage 2/2, cage 4/5, cage 5/1, cage 5 / 2, N9 and Q3) tested positive (Table 1). To mitigate the potential for TB outbreaks, we conducted investigations involving 100 cynomolgus macaques housed in 11 cages at 3, 6 and 12 months intervals. At 3 and 6 months later, an additional 4 (4.0%) and 2 (2.1%) cynomolgus macaques, respectively, tested positive and were subsequently moved to individual cages. After 12 months, only 1 (1.1%) positive cynomolgus macaque was detected. It should be noted that positive cases were mainly concentrated in just 2 cages (C5/1 and Q1) during the third month, and no further positive cages were detected until the 12-month mark (Fig. 2). In the absence of a rapid screening assay, the manifestation of clinical signs occurred over a span of 4–15 months (Table 1). Moreover, depending on the traditional TB culture investigation, which can yield results in 1–3 months. Our findings also demonstrated that the Xpert MTB/RIF Ultra assay always provided faster results than culture due to contamination from rapidly growing bacteria, nontuberculous mycobacteria, or exposure to bacteriostatic agents during sample collection or processing, resulting in the absence of growth of *M. tuberculosis* (Table 1 and Fig. 2). This delay in detection could potentially lead to outbreaks not only between animals, but also between staff.

## Discussion

Cynomolgus macaques, along with other macaque species, are susceptible to *Mycobacterium tuberculosis*, the causative agent of human tuberculosis (TB)^2,15^. This susceptibility raises significant concerns about the potential transfer of the pathogen between nonhuman primates and humans^5,11^. Therefore, the detection and effective management of MTBC infection in cynomolgus macaques are crucial for preventing zoonotic transmission, ensuring the well-being of both macaque populations and human communities, and facilitating continued research efforts in macaques to gain insight into TB pathogenesis, immunity, vaccine development, and treatment^16^.

Currently, TB diagnosis in macaques is a complex and time-consuming process due to challenges such as the multifaceted diagnostic approach, the potential for cross-reactivity in tests, the limited sensitivity in disease detection, and the difficulties in collecting high quality samples, highlighting the need for ongoing efforts to improve diagnostic methods^2,4,6,11,17^. Today, the diagnosis and detection of MTBC infection in cynomolgus macaques often involve molecular tools similar to those used in the diagnosis of human TB, such as conventional polymerase chain reaction (PCR)^2^, digital PCR (dPCR)^18^ , real-time PCR (qPCR), nested PCR^11^, and fluorescence hybridization in situ (FISH)^19^. However, the results of these diagnostic tests may exhibit variability between laboratories. This variability may be attributed to an array of factors, including disparities in the composition of reagents, variations in staff performance, and the use of distinct laboratory equipment. The Xpert MTB/RIF Ultra test can be used with various types of clinical specimens and is considered easy and fully automated^20,21^, making it a valuable tool to diagnose TB in cynomolgus macaques. Prior studies have documented successful applications of this human diagnostic test for TB detection in livestock and wildlife animals^22–24^. However, our study represents the first investigation to unveil the potential of the Xpert MTB/RIF Ultra assay as a first-line technique to significantly enhance the speed and accuracy of diagnosing tuberculosis in macaques.

The combined results of the Xpert MTB/RIF Ultra assay with conventional TB culture demonstrated comparable detection performance to the Xpert MTB/RIF Ultra assay alone, highlighting the superiority of the Xpert MTB/RIF Ultra assay in the field of animal diagnostics (Table 1). It is crucial to highlight that our results did reveal culture-positive nontuberculous mycobacteria (NTM) in samples that tested positive with the Xpert MTB/RIF Ultra assay, including *M. intracellulare, Mycobacterium* sp*., M. fortuitum* and *M. scrofulaceum*, instances of cross-reactivity with these NTM species leading to false positive TB results have been rarely reported, except for *M. scrofulaceum*, which occurred at particularly high bacterial loads (10^8^ CFU/mL)^9,25,26^. It is essential to note that such elevated concentrations are typically associated with artificially generated nonhuman samples and are not commonly encountered in natural settings. Furthermore, NTMs were also cultured in 24 of the 298 negative Xpert MTB/RIF Ultra macaque samples (8.05%) (annex1). This occurrence could potentially be attributed to the exposure of animals to NTM-containing sources such as water, soil, and other environmental materials^27^ without sign and symptom of disease. Our findings demonstrate the detection capacity of the Xpert MTB/RIF Ultra assay and suggest that cross-reactivity between MTB and five species of NTM may not be a systematic issue at the animal level. As well as, various factors, including the presence of other bacteria, TB death during transportation, sampling errors, and the strength of decontamination solutions, can contribute to limitations in TB culture results. These factors may lead to eccentric or unexpected results in the screenings for animal no. 91, 100, 103, and 293 (Supplementary Information).

The Xpert MTB/RIF Ultra test can be used with various types of clinical specimens^28^. This versatility is crucial when diagnosing tuberculosis in different contexts, including in animals such as cynomolgus macaques. In macaque research settings, especially for the molecular diagnosis of TB, common specimens used for testing typically include non-sputum samples. Especially upper respiratory tract sampling holds promise to expand access to TB diagnosis, including oral sampling and larynx and nasopharynx sampling^29^. PW specimens are typically collected from areas where MTBC bacteria are most likely to be present in cases of pulmonary TB. On the other hand, BS specimens are oral sampling and one of the common sites of MTBC shedding, making it a likely less invasive specimen collection method, and it has shown promising results^11,30,31^. The current data highlights the sensitivity and efficacy of PW samples compared to BS samples using the Xpert MTB/RIF Ultra test, as evidenced by the definitive identification of tuberculosis infection in three of five positive cases.

The turnaround time for mycobacterial cultures typically ranges from 2 to 6 weeks for the reporting of results. Infectiousness and duration of infectiousness can be reduced through early case detection of active case finding and linkage by using quicker and more sensitive diagnostic assays^32^. Our study provides compelling evidence of how the rapid turnaround time of the Xpert MTB / RIF Ultra assay and prompt isolation measures facilitate the rapid isolation and treatment of infectious individuals in a single day, significantly reducing the risk of TB transmission in communities, healthcare facilities, and captive animal populations (Fig. 2). One limitation of this study is the lack of precise information on the timeline of initial infection and the ability to identify index cases. Despite the implementation of prompt isolation measures for cynomolgus macaques testing positive for TB, it is imperative to recognize that the interval between exposure to TB bacteria and the onset of active disease can extend from months to years. As demonstrated in our study, these measures contributed to a reduction in positive cases among the macaque population, suggesting the effectiveness of the cascade reduction approach. Further studying whole-genome sequencing (WGS) in macaque TB isolates is the ability to gain deep insights into TB biodiversity and can improve knowledge on pathogenesis, virulence, drug resistance, and transmission dynamics in a controlled research setting which benefits both TB research and efforts to develop new diagnostics, treatments, and preventive measures for TB in both humans and other animals.

In addition to its rapidity and a limit of detection (LOD) of 16 units of bacterial colony formation (cfu) per ml, this assay offers valuable information on RIF susceptibility or resistance^8,9^. Among the isolates obtained from animals numbered 49, 55, 91, 99, 100, 103, and 293, at least 7 of 18 (38.9%) were found to be susceptible to RIF. However, it is important to mention that at very low bacterial loads, resulting in a "trace" result (n = 11, 61.1%) in our study, amplification of the *rpo*B target does not provide information regarding RIF susceptibility or resistance (Table 1). Notably, no MTBC RIF-resistant isolates derived from cynomolgus macaques were reported in this study.

## Conclusions

These findings elucidate the advancement of the Xpert MTB/RIF Ultra assay in TB diagnosis and its crucial role in preventing potential outbreaks in cynomolgus macaques. The rapidity, high sensitivity, and specificity of the assay make it suitable for nonhuman primate reference laboratories to confirm TB disease and interrupt the transmission of TB, particularly in research and wildlife management contexts where TB management is a significant health concern. It is worth performing Xpert annually to detect tuberculosis early in a suspected group of cynomolgus macaques and separate them from the cage group at the Wildlife Breeding Center.

### Supplementary Information

Supplementary Information.

## Data Availability

The datasets used and analysed during the current study are available from the corresponding author on reasonable request.
